# Nephrology Partnership for Advancing Technology in Healthcare (N-PATH) program: the teachers’ perspective

**DOI:** 10.1093/ckj/sfad299

**Published:** 2023-12-08

**Authors:** Carlo Lomonte, Michele Rossini, Jose Ibeas, Mauro Forcella, Jadranka Buturovic Ponikvar, Maurizio Gallieni, Roberto Russo, Dimitrios Goumenos, Vladimir Tesar, Zdenka Hruskova, Joris Roelofs, Sandrine Florquin, Maarten Snoeijs, Antonio Giusto, David Shemesh, Joris Rotmans, Roser Torra, Christoph Wanner, Loreto Gesualdo

**Affiliations:** Nephrology Unit, “F. Miulli” General Hospital, Acquaviva delle fonti, Bari, Italy; Renal, Dialysis and Transplantation Unit, Department of Precision and Regenerative Medicine and Ionian Area (DIMEPRE-J), University of Bari, Bari, Italy; Nephrology Department, Parc Taulí Hospital Universitari, Institut d'Investigació i Innovació Parc Taulí (I3PT-CERCA), Universitat Autònoma de Barcelona, Sabadell, Barcelona, Spain; Department of Nephrology, University Hospital “Ospedali Riuniti”, Foggia, Italy; Department of Nephrology, University Medical Centre Ljubljana and Faculty of Medicine, University of Ljubljana, Ljubljana, Slovenia; Department of Biomedical and Clinical Sciences, University of Milano, Milan, Italy; Renal, Dialysis and Transplantation Unit, Department of Precision and Regenerative Medicine and Ionian Area (DIMEPRE-J), University of Bari, Bari, Italy; Department of Nephrology and Renal Transplantation, University Hospital of Patras, Patras, Greece; Department of Nephrology, General University Hospital in Prague and First Faculty of Medicine, Charles University, Prague, Czech Republic; Department of Nephrology, General University Hospital in Prague and First Faculty of Medicine, Charles University, Prague, Czech Republic; Department of Pathology, Amsterdam University Medical Center, Amsterdam, The Netherlands; Department of Pathology, Amsterdam University Medical Center, Amsterdam, The Netherlands; Department of Vascular Surgery, Maastricht University Medical Center, Maastricht, The Netherlands; Renal, Dialysis and Transplantation Unit, Department of Precision and Regenerative Medicine and Ionian Area (DIMEPRE-J), University of Bari, Bari, Italy; Vascular Access Society, Department of Surgery and Hemodialysis Access Center, Shaare Zedek Medical Center, Jerusalem, Israel; Vascular Access Society, Leiden University Medical Center, Leiden, The Netherlands; European Renal Association, Nephrology Department, Fundació Puigvert, Institut de Recerca Sant Pau, Universitat Autònoma de Barcelona, Barcelona, Spain; European Renal Association, University of Würzburg, Department of Clinical Research and Epidemiology, Würzburg, Germany and University of Oxford, Nuffield Department of Population Health, CTSU, Oxford, UK; Renal, Dialysis and Transplantation Unit, Department of Precision and Regenerative Medicine and Ionian Area (DIMEPRE-J), University of Bari, Bari, Italy

**Keywords:** educational, hands-on training, interventional nephrology, N-PATH, simulation training

## Abstract

The N-PATH (Nephrology Partnership for Advancing Technology in Healthcare) program concluded with the 60th European Renal Association 2023 Congress in Milan, Italy. This collaborative initiative aimed to provide advanced training in interventional nephrology to young European nephrologists. Funded by Erasmus+ Knowledge Alliance, N-PATH addressed the global burden of chronic kidney disease (CKD) and the shortage of nephrologists. CKD affects >850 million people worldwide, yet nephrology struggles to attract medical talent, leading to unfilled positions in residency programs. To address this, N-PATH focused on enhancing nephrology education through four specialized modules: renal expert in renal pathology (ReMAP), renal expert in vascular access (ReVAC), renal expert in medical ultrasound (ReMUS) and renal expert in peritoneal dialysis (RePED). ReMAP emphasized the importance of kidney biopsy in nephrology diagnosis and treatment, providing theoretical knowledge and hands-on training. ReVAC centred on vascular access in haemodialysis, teaching trainees about different access types, placement techniques and managing complications. ReMUS recognized the significance of ultrasound in nephrology, promoting interdisciplinary collaboration and preparing nephrologists for comprehensive patient care. RePED addressed chronic peritoneal dialysis, offering comprehensive training in patient selection, prescription, monitoring, complications and surgical techniques for catheter insertion. Overall, N-PATH's strategy involved collaborative networks, hands-on training, mentorship, an interdisciplinary approach and the integration of emerging technologies. By bridging the gap between theoretical knowledge and practical skills, N-PATH aimed to revitalize interest in nephrology and prepare proficient nephrologists to tackle the challenges of kidney diseases. In conclusion, the N-PATH program aimed to address the shortage of nephrologists and improve the quality of nephrology care in Europe. By providing specialized training, fostering collaboration and promoting patient-centred care, N-PATH aimed to inspire future nephrology professionals to meet the growing healthcare demands related to kidney diseases and elevate the specialty's status within the medical community.

## INTRODUCTION

**Figure 1:**
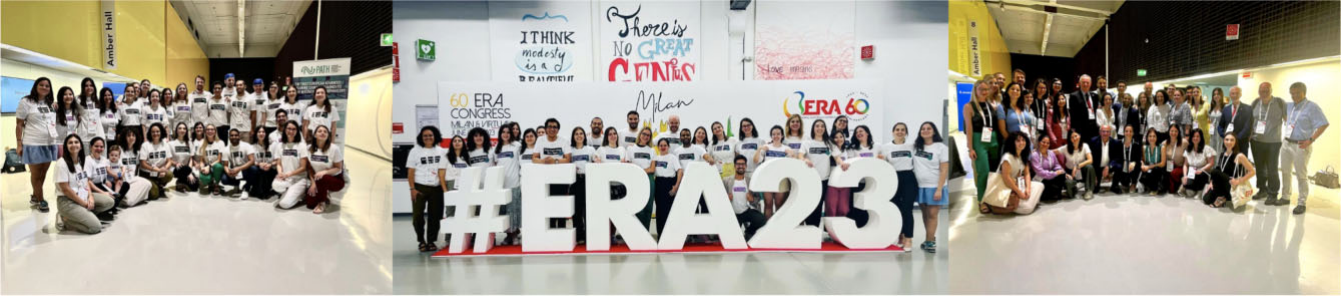
N-PATH during the 60th ERA Congress in Milan, Italy.

**Figure 2:**
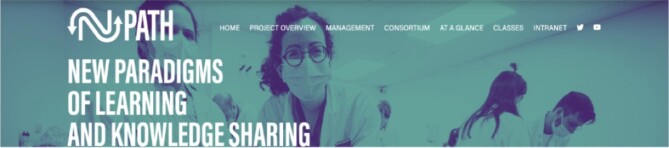
N-PATH website.

The N-PATH (Nephrology Partnership for Advancing Technology in Healthcare) program was a collaborative initiative aimed at providing advanced training in interventional nephrology to young European nephrologists. The program was officially concluded during the diploma ceremony at the 60th European Renal Association 2023 Congress in Milan, Italy (Fig. 1). The primary objective of N-PATH was to train 40 young European nephrologists in both theoretical knowledge and practical skills related to interventional nephrology. The training program was formally started with the kick-off meeting held in Parma, Italy, on 24 September 2021. The project's mission aligned with the European Union's goals of sharing knowledge and fostering collaboration between higher education institutions, small and medium enterprises and other stakeholders (European medical organizations) involved in the field of nephrology and interventional nephrology. N-PATH (https://npath.eu/; Fig. 2) was a consortium comprising eight prominent universities, two scientific societies and two companies. The participating universities included Amsterdam University Medical Centers, Università degli studi di Bari Aldo Moro, Barcelona Parc Taulí Hospital Universitari, University Medical Centre Ljubljana, Maastricht University Medical Center, Università degli studi di Milano, University of Patras and the General University Hospital in Prague. The European scientific societies involved were the European Renal Association (ERA) and Vascular Access Society (VAS). The two companies were EUREKA and EMAC funded by Erasmus+ Knowledge Alliance, a European Commission program (https://erasmus-plus.ec.europa.eu/), N-PATH was the first European-wide advanced training course in diagnostic and interventional nephrology. The University of Bari Aldo Moro, Italy, led the program under the leadership of its Department DiMePRe-J. The N-PATH program enhanced the expertise and skills of young nephrologists in interventional nephrology, fostered collaboration and networking among professionals and institutions and contributed to the advancement of nephrology care and technology in Europe [1].

### The importance of the N-PATH project

The N-PATH project was initiated in response to the significant global burden of chronic kidney disease (CKD), which affects >850 million people worldwide and ranks as one of the most prevalent non-communicable chronic degenerative diseases with high cardiovascular mortality [2]. In comparison with other well-known diseases like diabetes mellitus, cancer and HIV/AIDS, CKD stands out as being twice as common as diabetes and having a much higher prevalence [2]. As CKD is a progressive condition often associated with known risk factors such as diabetes and hypertension, it can ultimately lead to end-stage renal disease, necessitating dialysis or transplantation for treatment [3, 4]. Early detection and diagnosis of CKD are crucial in managing the disease and slowing its progression to more advanced stages [5]. Despite the growing prevalence of kidney diseases and the increasing demand for nephrology care, there is a concerning trend of nephrology not being seen as an attractive career choice for medical students and doctors in training. The specialty is facing challenges in recruiting new talent, leading to a shortage of nephrologists to address the healthcare burden imposed by kidney diseases [6, 7]. This lack of interest in nephrology is evident not only in the USA, but also in Europe, with many nephrology residency programs in 2021–2022 experiencing unfilled positions [8, 9]. We are faced with a new nephrological syndrome: acute fellowship insufficiency [6, 7].

Why do residents not like the nephrology as a specialty? Why is nephrology not attractive? To tackle these issues and improve the state of nephrology, the N-PATH project was conceived to create a specialized training program in diagnostic and interventional nephrology. By offering theoretical and practical training to young European nephrologists, the project aimed to enhance their skills and expertise in this field. Additionally, by promoting networking and collaboration between higher education institutions, universities, scientific societies and small enterprises, the program sought to increase interest and engagement in nephrology as a specialty. Through this project, the hope was to attract more talented individuals to nephrology and address the growing healthcare burden posed by kidney diseases across Europe and beyond.

### Unmet needs in nephrology education

The prediction of reductions in the nephrology workforce over the next decade, as indicated by global expert groups [10–12], raises concerns about the potential serious implications for healthcare systems and patients. While the situation varies from country to country, some common factors contribute to the shortage of nephrologists and declining interest in the specialty:

- Lack of exposure: Many medical students and residents do not receive sufficient exposure to nephrology during their training. As a result, they may not fully understand the scope and importance of the specialty, leading to fewer individuals considering it as a career option.
- Decline of interest: Nephrology may be perceived as less attractive or less prestigious compared with other specialties, which can lead to a decline in interest among medical trainees. The reasons behind this decline may include perceptions of limited career opportunities or a lack of recognition for nephrologists’ contributions to healthcare.
- Practical training: Trainees often seek more practical and hands-on experiences during their education. If nephrology training programs do not offer enough opportunities for practical training and clinical experiences, it may deter potential candidates from pursuing the specialty.
- Lack of mentors: Effective mentorship is crucial in nurturing interest and passion for a specialty. A shortage of experienced and supportive mentors in nephrology can impact the career choices of young medical professionals.
- Competition from other specialties: Nephrology faces competition from other medical specialties, such as cardiology and radiology, which may be perceived as more appealing or offering better career prospects.
- Geographic disparities: Disparities in the distribution of nephrologists across different countries can lead to imbalances in healthcare access. Some countries may have a higher concentration of nephrologists per million inhabitants, while others experience a shortage.

Addressing these challenges requires concerted efforts from medical education institutions, professional societies and healthcare policymakers. Measures that can be taken to attract and retain nephrologists include:

- Enhancing exposure to nephrology during medical education through well-structured curricula and clinical rotations.
- Creating opportunities for practical training and clinical experiences in nephrology.
- Establishing mentorship programs to support and guide medical trainees interested in nephrology.
- Raising awareness about the importance of nephrology and the rewarding aspects of the specialty.
- Implementing strategies to ensure a more equitable distribution of nephrologists across regions and countries.

By addressing these factors, it is possible to encourage more medical professionals to pursue nephrology as a career and meet the growing healthcare demands related to kidney diseases.

### N-PATH strategy to recruit excellent fellows in nephrology

N-PATH's strategy for recruiting excellent fellows in nephrology was comprehensive and well-structured, addressing key aspects of revitalizing interest in the specialty and preparing the next generation of nephrologists. The strategies that N-PATH implemented to recruit excellent fellows in nephrology were:

- Collaborative networks: N-PATH established a strong collaborative network involving European medical schools, scientific societies (ERA and VAS) and small and medium enterprises (EMAC and EUREKA). This network promotes nephrology as an attractive career choice, develops a standardized core curriculum and spreads knowledge through wider networks.
- Clinical skills and procedural training: The program offered hands-on training in essential procedural skills such as ultrasound, kidney biopsy, peritoneal dialysis (PD) and vascular access. Practical training with skilled tutors and cutting-edge simulators enhanced clinical proficiency and fostered the development of expertise specific to nephrology.
- Mentorship and teaching: N-PATH recognized the importance of excellent teaching and mentorship. By emphasizing mentorship, the program provided guidance and support to fellows throughout their nephrology career journey.
- Interdisciplinary approach: The program promoted a patient-centred and interdisciplinary approach to nephrology training, encouraging collaboration with other medical specialties and emphasizing holistic patient care.
- Research and educational facilities: N-PATH offered access to research and educational facilities that exposed fellows to the latest advancements in nephrology and provided opportunities for cutting-edge research.
- Incorporating emerging technologies: N-PATH recognized the importance of training in emerging technologies, including artificial intelligence, virtual and augmented reality, immersive reality, simulation and navigation, preparing fellows for the evolving landscape of medical practice.
- Structured curricula: N-PATH's four macro-topics curricula [renal expert in renal pathology (ReMAP), renal expert in vascular access (ReVAC), renal expert in medical ultrasound (ReMUS) and renal expert in peritoneal dialysis (RePED)] provided specialized training, ensuring a well-rounded education for fellows and catering to diverse interests within nephrology.
- Blended learning approach: N-PATH utilized a blended learning approach, combining face-to-face interactions, online learning, hands-on training and virtual and augmented reality tools. This approach maximized engagement and accessibility for trainees.
- Open-access e-learning platform: By releasing an open-access e-learning platform (https://npath.eu/login/), N-PATH was able to enhance knowledge dissemination and accessibility, reaching a broader audience of potential nephrology professionals.
- Academic certification: N-PATH awarded an Academic Certificate with 29 European Credit Transfer and Accumulation System (ECTS) to 40 fellows upon completion of the program, providing formal recognition of their training and expertise.
- Promotion of nephrology careers: N-PATH's efforts have contributed to promoting nephrology careers at the European Union (EU) level, making the specialty more appealing and inspiring the next generation of nephrologists.

**Figure 3:**
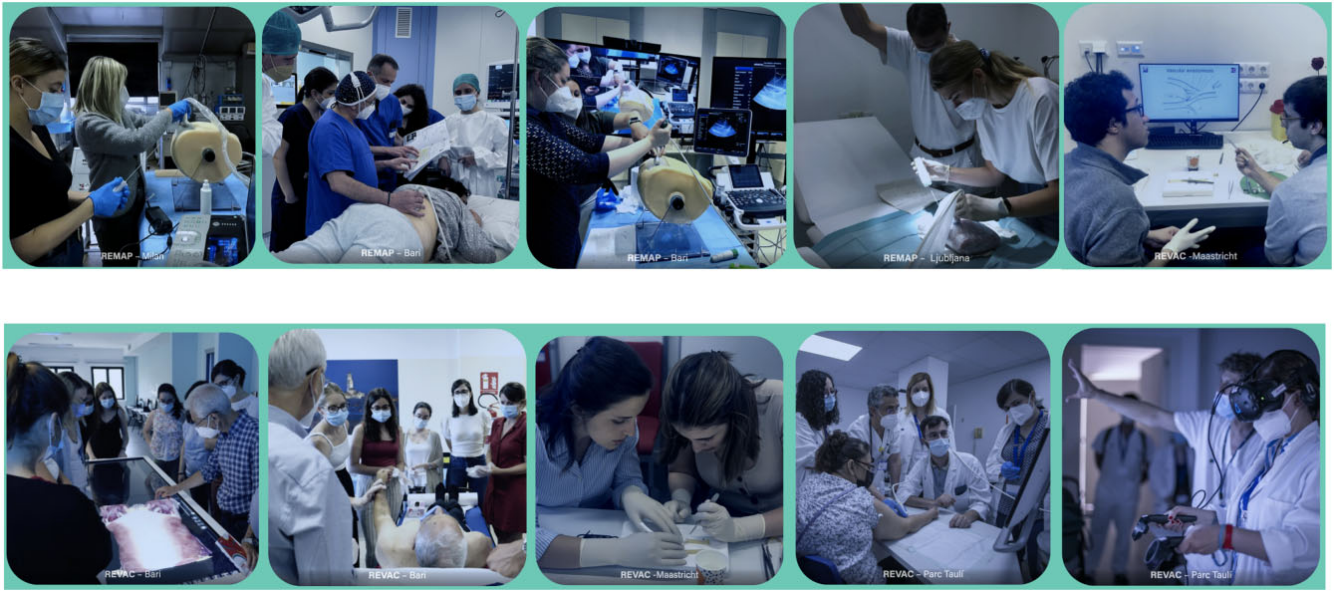
N-PATH training approaches.

Overall, N-PATH's strategy was not only comprehensive, but also adaptable to the changing demands of the medical field. By focusing on practical skills, mentorship, interdisciplinary collaboration and the integration of emerging technologies, N-PATH has effectively addressed the decline in interest in nephrology and created a community of engaged and skilled nephrology professionals (Fig. 3).

### ReMAP

ReMAP within the N-PATH program was focused on the kidney biopsy [13, 14]. This module recognized the historical significance and evolving importance of kidney biopsy in the field of nephrology and provided specialized training to nephrology fellows. The detailed overview of the ReMAP module's objectives, content and training approach are summarized below.

#### Objectives

Kidney biopsy has played a pivotal role in the development of nephrology, serving as a fundamental tool for accurate diagnosis and treatment of both acute and chronic renal diseases. Its significance is underscored by its ability to alter clinical diagnoses, guide treatment decisions and uncover pathogenic mechanisms underlying renal disorders. The ReMAP module highlights how histological diagnosis from kidney biopsy results in substantial clinical impact. It influences treatment strategies by determining lesion activity and reversibility and guiding therapy adjustments. Furthermore, kidney biopsy contributes to our understanding of disease pathogenesis, paving the way for novel therapeutic interventions. The ReMAP module emphasizes the crucial role of nephrologists in performing renal biopsies. While the procedure may be delegated to other specialists, the nephrologist's unique position as the primary caregiver of kidney disease patients allows for a comprehensive assessment of risks and benefits. This holistic perspective ensures the optimal balance between obtaining sufficient tissue for accurate diagnosis and minimizing potential complications.

#### Content and training approaches

The ReMAP module offered theoretical lessons through an e-learning platform. An international faculty panel imparted knowledge on technical aspects of kidney biopsy, histopathology, molecular pathology and the application of artificial intelligence to digital pathology. A 4-day hands-on program immersed trainees in practical aspects. They engaged in discussions centred around real patient cases, refining the approach to kidney biopsy for different patient phenotypes. Trainees observed renal biopsies and learned post-procedure patient management, including recognizing and managing complications. Trainees gained practical experience using both physical simulators (dedicated mannequin models) and virtual simulators. This allowed them to practice performing renal biopsies, enhancing their technical skills and procedural proficiency. Trainees participated in discussions involving real clinico-pathological cases. This fostered a deeper understanding of applying histopathology and molecular pathology techniques to the study of kidney biopsy.

#### Outcome and ultimate goal

By the end of the ReMAP module, trainees were able to:

- Identify indications and contraindications for kidney biopsy.
- Perform kidney ultrasound examinations and select appropriate approaches for kidney biopsy based on patient phenotypes.
- Understand technical aspects, such as needle size and patient positioning.
- Manage potential complications associated with kidney biopsy.
- Handle tissue obtained from kidney biopsy effectively.
- Apply classical and novel histology and molecular pathology techniques to the study of kidney biopsy.

In summary, the ReMAP module within N-PATH's training program was designed to equip nephrology fellows with the expertise and skills necessary for performing renal biopsies. By providing both theoretical knowledge and hands-on training, the module ensured that trainees were well-prepared to make accurate diagnoses, guide effective treatments and contribute to advancing the field of nephrology through research and patient care.

### ReVAC

The ReVAC program of the N-PATH initiative focused on the crucial aspect of vascular access in nephrology practice, particularly for patients on haemodialysis (HD) [15–21]. Recognizing the significant impact of vascular access–related issues on patient outcomes, healthcare costs and overall patient well-being, the ReVAC program aimed to equip nephrology fellows with comprehensive knowledge and skills related to vascular access management. Below is a detailed overview of the ReVAC program's objectives, content and training approach.

#### Objectives

Patients on HD often encounter challenges and complications related to vascular access. Nephrologists play a pivotal role in managing these issues, as they significantly impact patient morbidity, mortality and healthcare costs. Nephrologists without a solid foundation in vascular access management may find themselves ill-equipped to address these critical concerns. The ReVAC program recognized the multidisciplinary nature of vascular access care. Collaboration with professionals such as vascular surgeons, interventional radiologists and HD nurses is essential to ensure comprehensive and effective management. Nephrologists, as primary caregivers, need to take a leading role in coordinating this interdisciplinary team.

#### Content and training approaches

The ReVAC program offered a 7-day hands-on course where trainees engaged with simulators and real patients to develop practical skills. Trainees learned how to perform physical examinations, make decisions for primary and secondary access and conduct ultrasound vascular mapping and surveillance. Moreover, they gained a thorough understanding of vascular anatomy, surgical dissection and endovascular techniques and the various routes for accessing the arterial and venous systems. This knowledge formed the basis for constructing arteriovenous accesses tailored to individual patient needs and its rescue when it fails. Trainees became proficient in identifying different catheter types, determining appropriate access sites and performing both non-tunnelled and tunnelled catheter placements using phantoms. The program addressed vascular access complications and provided insights into effective approaches for their treatment. This included discussions on complications and strategies to manage them. Finally, the trainees explored patients’ eligibility and indications for endovascular arteriovenous fistula creation, highlighting the importance of an interdisciplinary vascular access teamwork approach. The ReVAC program employed effective teaching methodologies, including demonstration and deliberate practice. Trainees learned through observing proper techniques demonstrated by tutors and engaging in mindful repetition of tasks with constructive feedback, leading to improved performance.

#### Outcome and ultimate goal

The ultimate goal of the ReVAC program was to provide trainees with a comprehensive understanding of vascular access care within a multidisciplinary model. By equipping nephrologists with the necessary knowledge and skills, the program aimed to enhance the effectiveness, efficiency and safety of patient treatment. Through this program, nephrology fellows were prepared to take on a leadership role in coordinating vascular access care, thus improving patient outcomes and reducing the burden of complications associated with HD vascular access [22]. In summary, the ReVAC program was designed to empower nephrology fellows with the expertise to manage vascular access–related challenges, ensuring that they enter practice well-prepared to address a significant source of patient problems and contribute to improved patient care within a multidisciplinary framework.

### ReMUS

The ReMUS module of the N-PATH program recognized the pivotal role of ultrasound in modern nephrology practice. Ultrasound has emerged as a fundamental tool in the hands of nephrologists, enabling efficient and integrated decision making at the patient's bedside—i.e. point-of-care ultrasound [23]. This module aimed to equip nephrology fellows with the knowledge and skills necessary to utilize ultrasound effectively in the diagnosis, therapeutic decisions and interventional procedures for patients with renal pathologies. The detailed overview of the ReMUS module's objectives, content and training approach are summarized below.

#### Objectives

Ultrasound has become an essential component of clinical practice for nephrologists, facilitating informed decision making by integrating clinical history, physical examination and dynamic ultrasound examination. This comprehensive approach leads to more efficient decision making and benefits both patients and healthcare systems by optimizing resource utilization. The ReMUS module recognizes the value of interdisciplinary collaboration, enabling nephrologists to work alongside other specialties in making diagnostic and therapeutic decisions. This collaborative approach enhances the quality of patient care and leads to more effective outcomes.

#### Content and training approaches

The ReMUS module comprised both basic and advanced concepts. Basic concepts covered the physical principles of ultrasound, usage of ultrasound machines, ultrasound anatomy of the kidney and urinary tract and identification of anatomical alterations. Advanced topics encompassed a wide range of renal and systemic conditions, including acute and chronic renal failure, cystic diseases, solid renal and urinary tract masses, renal artery pathology, transplanted kidney and more. The module combined e-learning lectures via web streaming with 6 days of hands-on training. This training allowed students to practically apply their knowledge and skills, enhancing their proficiency in using ultrasound for comprehensive renal patient assessment. The ReMUS module's concepts were interconnected with other modules, including vascular access, PD and kidney biopsy. This integration ensured a holistic understanding of ultrasound's role in various aspects of nephrology. Trainees acquired in-depth knowledge and practical skills for effectively using ultrasound in diagnosing and managing renal patients. This included understanding the physical principles of ultrasound, interpreting ultrasound images and identifying relevant anatomical and pathological features. The module also sought to raise awareness among trainees about their role in a generational change in medical practice within nephrology. By integrating ultrasound into their practice, nephrologists may contribute to more comprehensive and effective patient care.

#### Outcome and ultimate goal

The ultimate goal of the ReMUS module was to empower nephrology fellows to confidently incorporate comprehensive ultrasound into their clinical practice. By doing so, they enhanced their diagnostic and therapeutic capabilities, contributed to interdisciplinary collaboration and paved the way for a new era of nephrology practice. In summary, the ReMUS module was designed to provide nephrology residents with the knowledge, skills and awareness necessary to harness the power of ultrasound in renal patient care. By bridging the gap between theory and practical application, the module supported the development of well-rounded nephrologists who can navigate complex clinical scenarios and contribute to advancements in nephrology practice.

### RePED

The RePED module of the N-PATH program addressed the essential components of establishing a successful chronic PD program. PD requires a comprehensive approach, involving trained physicians, safe access to the peritoneal cavity and thorough knowledge of clinical management [23–26]. The RePED module aimed to bridge the knowledge gap and equip nephrologists with the skills necessary to effectively manage PD patients. Here is an overview of the RePED module's objectives, content and training approach.

#### Objectives

Nephrologists need to be well-versed in the principles and clinical management of PD. The RePED module ensured that physicians are adequately trained to provide optimal care to PD patients. Permanent and safe access to the peritoneal cavity is crucial for successful PD. The module covered the surgical techniques of catheter insertion, addressing complications and ensuring the longevity of the access. Low utilization of PD by nephrologists can be attributed to inadequate training during nephrology residency. The RePED module aimed to fill this knowledge gap and provided comprehensive training to practicing nephrologists.

#### Content and training approaches

The RePED module included lectures that covered fundamental medical aspects of PD as a renal replacement therapy. Topics ranged from patient selection and peritoneal membrane physiology to treatment prescription, monitoring, complications, adequacy evaluation and long-term management.

Lectures were complemented by discussions of clinical cases and surgical techniques. Trainees gained insights into critical tasks such as catheter placement, removal and management of complications. Moreover, they received hands-on training in catheter insertion techniques. Various placement methods were described and practiced, including surgical placement by dissection, laparoscopy and blind placement using a guidewire. Finally, trainees interacted with an advanced, realistic dummy simulator that mimicked the anatomy of the peritoneal cavity and abdominal wall. The simulator provided different tissue types and dissection planes, allowing guided learning for catheter placement procedures under the supervision of a tutor. Using the simulator, trainees acquired operational skills and techniques necessary for catheter insertion, as well as practice with patients, experience in the use of ultrasound for the preparation and placement of the peritoneal catheter.

#### Outcome and ultimate goal

Trainees acquired skills in comprehensively managing PD patients. This included understanding the physiological aspects, prescription, monitoring, complications and long-term care of PD patients. The module ensured that trainees gained proficiency in the insertion and surgical techniques for accurate catheter placement. This aspect is crucial for ensuring safe and effective PD. In summary, the RePED module was designed to empower nephrology fellows with the knowledge and skills necessary for successful chronic PD programs. By covering essential aspects of PD patient management and providing hands-on training with advanced simulators and patients, the module contributed to improving patient care, reducing complications and enhancing the overall quality of nephrology practice.

### Trainees’ satisfaction

As reported in the participant feedback survey, the quality and comprehensiveness of the training delivered during each N-PATH module were beyond their expectations. They considered the e-lectures educational content adequate for understanding the topics covered in the exam, the meet-the-expert sessions as a unique opportunity to interact with the teachers and stimulate collaborative activities and, finally, they judged the hands-on sessions as a learning environment to practice skills in repetition with an experienced tutor providing feedback [27]. A summary of the strengths, weaknesses, opportunities and threats of the N-PATH project was also reported (Supplementary Fig. S1).

## CONCLUSION

In conclusion, the field of nephrology faces the important task of redefining its value and relevance in the management of kidney-related issues. It is essential to recapture the dynamic and clinically challenging spirit that has characterized the specialty in the past. Interventional nephrology presents both an opportunity and a challenge, particularly when built upon the foundation laid by pioneers in the field [28]. A crucial step toward this revitalization is the promotion of multidisciplinary education in interventional nephrology among trainees and young nephrologists. Academic institutions and nephrology residency programs play a vital role in ensuring high-quality healthcare and research in this domain. Details on the N-PATH project, topics of the web lessons and the hands-on program can be found on websites [1, 29]. The emerging generation of interventional nephrologists will take on increasingly central roles, leading multidisciplinary teams and overseeing intricate and potentially risky procedures. In this context, patient safety becomes paramount. Nephrologists must be adept at engaging patients in shared decision making, drawing on both research evidence and the unique perspectives of each patient [30]. By embracing these principles and equipping nephrologists with advanced skills and a patient-centric approach, the specialty can not only elevate its standing within the medical community, but also attract a new generation of enthusiastic physicians. The challenges of interventional nephrology can be transformed into opportunities for growth, innovation and improved patient outcomes. The N-PATH program offers an innovative educational model towards a new nephrological horizon, and as a proposal for the future, a memorandum of understanding already shared between interested and allied organizations in the N-PATH consortium will be signed, which will propose the model as a training standard.

## Supplementary Material

sfad299_Supplemental_File
