# Supplementary figures and images for: Nephrology Partnership for Advancing Technology in Healthcare (N-PATH) program: the teachers’ perspective
Source: Clin Kidney J. 2023 Dec 8;17(1):sfad299. doi: 10.1093/ckj/sfad299 (PMC10783234; doi:10.1093/ckj/sfad299)

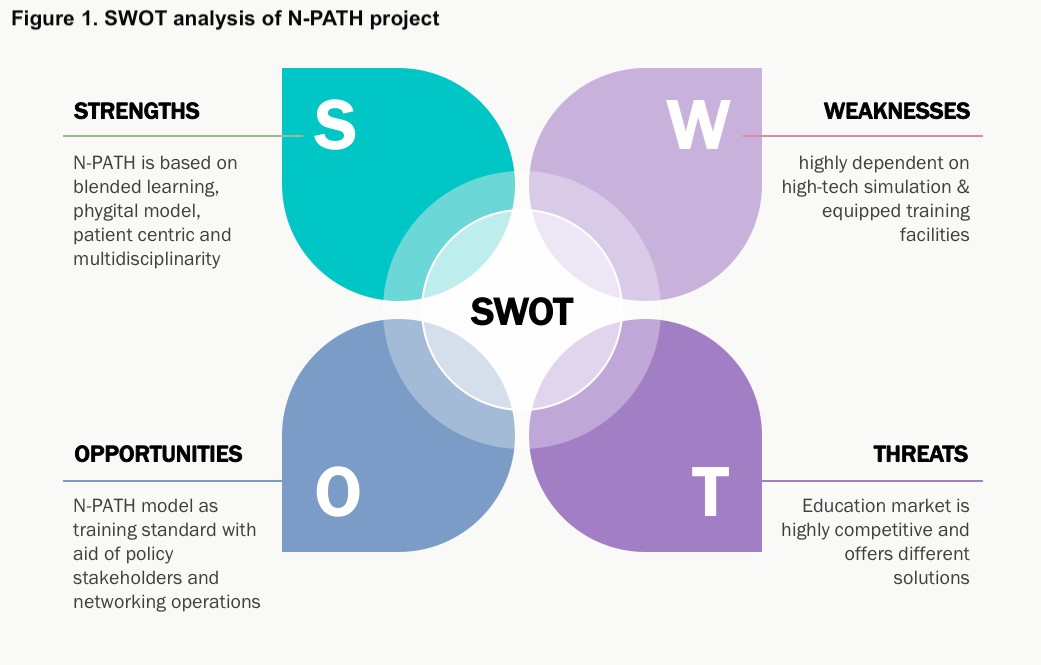

Supplement: sfad299_Supplemental_File [file sfad299_supplemental_file.jpeg]
